# Supplementary material for: Treatment Regimens for Immunocompetent Elderly Patients with Primary Central Nervous System Lymphoma: A Scoping Review
Source: Cancers (Basel). 2021 Aug 24;13(17):4268. doi: 10.3390/cancers13174268 (PMC8428349; doi:10.3390/cancers13174268)
Supplement: Supplementary file 1 [file cancers-13-04268-s001.zip › cancers-1305071-supplementary.pdf]

Table S1 Characteristics of retrospective studies

Study	Title	Population	Treatment	Number of patients
Farhi 2018[89]	Impact of front line relative dose intensity for MTX and comorbidities in immunocompetent elderly PCNSL patients	Immunocompetent patients ≥ 60 years diagnosed with a histology-proven DLBCL subtype of PCNSL (by stereotactic biopsy or open surgery) who received first-line treatment including at least 1 infusion of HD-MTX.	Comparison of 4 different HD-MTX-based treatment protocols.	N= 35
Han 2017[90]	Efficacy and safety of HD-MTX based systemic chemotherapy regimens: retrospective study of induction therapy for PCNSL in Chinese	Adult patients (range, 17–78) with newly diagnosed PCNSL.<br>(Age subgroup analyses available).	Comparison of the efficacy and safety of different HD-MTX based systemic chemotherapy regimens as induction therapy.	N= 47<br>> 60 years: N=18
Martinez-Calle 2020[85]	Outcomes of older patients with PCNSL treated in routine clinical practice in the UK: MTX dose intensity correlates with response and survival	Patients aged 65 years or older with a confirmed diagnosis of PCNSL (including vitreo-retinal lymphoma). Radiological exclusion of systemic disease by cross-sectional imaging at presentation was mandatory.<br>Exclusion criteria included surgically managed patients, solid organ transplant-related lymphoma and post-mortem diagnosis; HIV-positive patients were not excluded.	Patients were categorised based on clinician's treatment choice into 1) palliative including WBRT, 2) less intensive: MTX± rituximab ± alkylators, and 3) intensive: MTX/AraC combinations.	N= 244
Nakasu 2016[91]	Response-adapted treatment with upfront HCT-ASCT or consolidation phase HD-MTX for PCNSL: a long-term mono-center study	Adults with histological diagnosis of PCNSL<br>(Age subgroup analyses available).	Patients were treated with HD-MTX.<br>High dose chemotherapy with ASCT as rescue for patients under 65 years of age, and HD-MTX consolidation therapy for patients who were 65 years and older.	N= 61<br>≥ 70 years: N= 27
Ney 2010[86]	Characteristics and outcomes of elderly patients with PCNSL: the Memorial Sloan-Kettering Cancer Center experience	Patients with histologically confirmed PCNSL, aged 65 or older at the time of diagnosis.	Treatment regimens included WBRT, or chemotherapy, or both.	N= 174
Schuurmans 2010[92]	PCNSL in the elderly: a multicentre retrospective analysis	Patients aged 60 or older who were HIV-negative and diagnosed with histologically proven non-Epstein-Barr virus -associated.	Treatment regimens included WBRT, or chemotherapy, or both.	N= 74
Xie 2013[93]	PCNSL in the elderly: the Cleveland clinic experience	Patients who were diagnosed and treated for PCNSL and were older than 60 years at the time of diagnosis at the Cleveland Clinic between January 1986 and December 2010.	Treatment regimens included WBRT, or chemotherapy with or without consolidation WBRT.	N= 54

Fliessbach 2003[94]	Cognitive performance and MRI findings after high-dose systemic and intraventricular chemotherapy for PCNSL	Patients with histologically proven PCNSL, who were negative for human immunodeficiency virus.<br>(Age subgroup analyses available).	Chemotherapy protocol consisting of MTX and AraC in combination with intravenous application of vinca-alkaloids and alkylating agents.	N= 20<br>> 60 years: N=5
Gaviani 2016[95]	Safety and efficacy of PCNSL treatment in elderly population	Immunocompetent patients older than 70 years, with histologically confirmed PCNSL, without systemic involvement, treated with HD-MTX.	HD-MTX.	N= 17
Gregory 2013[96]	Rituximab is associated with improved survival for aggressive B cell CNS lymphoma	Cases of primary DLBCL of the CNS treated at the participating institutions in Victoria, Australia, during 1996 –2011. Patients were included if they were HIV negative and had a first presentation of biopsy-proven CD20+ primary DLBCL affecting the CNS.<br>(Age subgroup analyses available).	Front-line chemotherapy regimens included single agent HD-MTX alone or HD-MTX and AraC or other combination chemotherapy with HD-MTX, or combination chemotherapy without HD-MTX.	N= 120<br>> 60 years: N= 76
Hayakawa 1994[87]	PCNSL in Japan--a retrospective, co-operative study by CNS-Lymphoma Study Group in Japan	Adult patients with non-Hodgkin's PCNLS, confirmed with histopathology (group 1) or neuroimaging alone (group 2), retrospectively analysed.<br>(Age subgroup analyses available).	Patients were treated with various treatment types including surgery, WBRT, chemotherapy (various regimens with single or combined agents).	N= 170<br>≥ 60 years: N= 133
Kasenda 2012[37]	The prognostic value of serum MTX area under curve in elderly primary CNS lymphoma patients	Immunocompetent patients aged 60 or older diagnosed with PCNSL. Criteria for analysis were complete documentation of at least 1 HD-MTX application plus folinic acid rescue and monitoring of kidney and liver function during the courses of treatment.	3 different HD-MTX based treatment protocols.	N= 49
LOC Study Houillier 2017[97]	Rituximab, MTX, procarbazine, vincristine and intensified AraC consolidation for PCNSL in the elderly**: a LOC network study**	Newly diagnosed PCNSL, histologically proven diffuse large B-cell lymphoma, age over 60 years, and measurable enhancing lesion for response assessment. Patients with isolated ocular lymphomas or immunodeficiency, including HIV-positive status, were excluded.	Rituximab, MTX, procarbazine, vincristine and intensified AraC consolidation.	N= 90
LOC Study Collignon 2019[98]	(R)-GEMOX chemotherapy for unfit patients with refractory or recurrent PCNSL: a LOC study	Retrospectively identified adult immunocompetent patients with refractory or recurrent PCNSL treated by (R)-GEMOX. Additional inclusion criteria consisted in histological confirmation of PCNSL, anatomopathology consistent with diffuse large B cell lymphoma, radiographic evidence of CNS involvement at recurrence, and absence of systemic dissemination.<br>(Data for each patient available).	R-GEMOX regimen (gemcitabine, oxaliplatine, rituximab).	N= 13<br>> 60 years: N= 11

LOC Study Houillier 2020[17]	Management and outcome of primary CNS lymphoma in the modern era: An LOC network study	Patients enrolled in the present study had to fulfill the following criteria: (1) PCNSL diagnosis from January 1, 2011, and thereafter; (2) pathologic or cytologic confirmed diagnosis; (3) negative full-body CT scan or FDG-PET scan; (4) age greater than 18 years; (5) immunocompetence and negative HIV status. (Age subgroup analyses available).	Various chemotherapy treatments, without or with consolidation treatment after induction chemotherapy, consisting of either WBRT or HCT-ASCT.	N= 1.002<br>> 60 years: N= 717
Lee 2014[99]	Prognosis factors in Japanese elderly patients with PCNSL treated with a nonradiation, intermediate-dose MTX-containing regimen	Patients aged 60 years or older treated with a modified version of the EORTC protocol. PCNSL was diagnosed by pathologists on the basis of histological and immunohistochemical studies according to the World Health Organization classification.	Modified version of the EORTC protocol (MTX, ranimustine, procarbazine, methylprednisolone, AraC, Leucovorin).	N= 38
Makino 2015[100]	Prognostic impact of completion of initial HD-MTX therapy on PCNSL: a single institution experience	Newly diagnosed adult immunocompetent PCNSL patients. PCNSL was histologically confirmed by biopsy or resection. (Age subgroup analyses available)	HD-MTX (all ages) and WBRT (only patients <60 years).	N= 91<br>≥ 60 years: N= 63
Madle 2015[101]	The influence of rituximab, HCT-ASCT, and age in patients with primary CNS lymphoma	Patients with PCNSL who were treated in the Heidelberg University Hospital between 2000 and 2011 at least once were included. (Age subgroup analyses available).	Multivariate analysis for Rituximab, ASCT, WBRT.	N= 81<br>> 60 years: N= 51
Omuro 2007[45]	Temozolomide and MTX for PCNSL in the elderly	Immunocompetent patients older than 60 with newly diagnosed PCNSL seen between January 2003 and April 2004 at 3 institutions from the French Association of Neuro-Oncology in Paris.	Treatment consisting of an induction phase with MTX and temozolomide. Leucovorin and hydration were given as per institutional guidelines.	N= 23
Sonoda 2007[102]	PCNSL treated with combined intra-arterial nimustine and WBRT	Newly diagnosed PCNSL patients who were treated between 1995 and 2005, at Miyagi Cancer Center Hospital. Their clinical data were retrospectively evaluated. (Age subgroup analyses available).	Combination therapy consisting of intra-arterial nimustine and WBRT.	N= 63<br>≥ 60 years: N= 33
Taoka 2010[103]	A nonradiation-containing, intermediate-dose MTX regimen for elderly patients with PCNSL	Consecutive patients with newly diagnosed PCNSL aged over 60 years, or aged 55–60 years if they had poor performance status. Stereotactic surgery or tumor resection under craniotomy was performed and diagnosis was made on the basis of histological and immunohistochemical studies	Modified protocol from EORTC Brain Tumor Group: MTX, ranimustine, pocarbazine, methylprednisolone.	N= 17

Welch 2012[84]	Outcomes of the oldest patients with PCNSL treated at Memorial Sloan-Kettering Cancer Center	Institutional database was used to identify patients with PCNSL who were diagnosed at 80 years or older between 1993 and 2011. Data were collected by chart review, and response was determined by contrast-enhanced MRI or CT.	MTX-based chemotherapy.	N= 24
**Studies specifically investigating HCT-ASCT (N=2)**				
Kassam 2017[88]	HCT-ASCT for PCNSL: a multi-centre retrospective analysis from the United Kingdom	Adult patients were identified from local bone marrow transplant databases. Patients were eligible if they had a biopsy proven diagnosis of PCNSL of DLBCL histology and had undergone a thiotepa-conditioned HCT-ASCT for PCNSL in first response. (Age subgroup analyses available)	HCT-ASCT**.**	N= 70<br>≥ 65 years: N= 22
Schorb 2017[30]	Thiotepa-based HCT-ASCT in elderly patients with PCNSL: a European retrospective study	Elderly patients aged 65 or older with histologically proven PCNSL, without systemic lymphoma manifestation, no evidence of immunodeficiency and completed thiotepa-based HCT-ASCT.	Thiotepa-based HCT-ASCT	N= 52

AraC: cytarabine; **CNS**: central nervous system; **DLBCL: d**iffuse large B cell lymphoma; **PET: p**ositronenemissionstomographie; **HD-MTX**: high-dose methotrexate; **HIV**: human immunodeficiency virus; **MRI**: magnetic resonance imaging; **PCNSL**: primary central nervous system lymphoma; **R-GEMOX**: gemcitabine, oxaliplatine, rituximab; **WBRT**: whole brain radiotherapy

Table S2: Systematic literature search (second search)

#	Searches	Results
1	(lymphom* adj3 cns).ti,ab,kf.	1919
2	(lymphom* adj3 brain).ti,ab,kf.	876
3	(lymphom* adj3 neuro*).ti,ab,kf.	914
4	(lymphom* adj3 nerv*).ti,ab,kf.	3062
5	(lymphom* adj3 centr*).ti,ab,kf.	4124
6	(lymphom* adj3 spin*).ti,ab,kf.	306
7	PCNSL.ti,ab,kf.	1521
8	((central nervous system or cns) adj3 (neoplasm$ or lymphom$ or tumor$ or tumour$)).ti,ab,kf.	13569
9	or/1-8	16498
10	exp Antineoplastic Agents/ or exp Antineoplastic Agents/ or exp Antineoplastic Combined Chemotherapy Protocols/ or Drug Therapy/ or exp Chemotherapy, Adjuvant/ or exp Consolidation Chemotherapy/ or exp Induction Chemotherapy/ or exp Maintenance Chemotherapy/ or exp Cytostatic Agents/	1189186
11	(chemotherap* or radiochemotherap* or ((anti neoplast* or antineoplast*) and (drug* or agent*))).ti,ab,kf.	431044
12	10 or 11	1392536
13	9 and 12	4568
14	exp aged/ or exp geriatrics/	3150990
15	(centarian* or centenarian* or elder* or eldest or frail* or geriatri* or nonagenarian* or octagenarian* or octogenarian* or old age* or older adult* or older age* or older female* or older male* or older man or older men or older patient* or older people or older person* or older population or older subject* or older woman or older women or oldest old* or senior* or senium or septuagenarian* or supercentenarian*).ti,ab,kf.	556054
16	("60 years" or "65 years" or "65-years" or "60-years" or "70 years" or "70-years" or "75 years" or "75-years" or "80 years" or "80-years" or "85 years" or "85-years" or "90 years" or "90-years" or "95 years" or "95-years" or "100 years" or "100-years" or "60 year" or "65 year" or "65-year" or "60-year" or "70 year" or "70-year" or "75 year" or "75-year" or "80 year" or "80-year" or "85 year" or "85-year" or "90 year" or "90-year" or "95 year" or "95-year" or "100 year" or "100-year" or "sixty years" or "sixty-years" or "sixty-five years" or "sixty-five-years" or "seventy years" or "seventy-years" or "seventy-five years" or "seventy-five-years" or "eighty years" or "eighty-years" or "eighty-five years" or "eighty-five-years" or "ninety years" or "ninety-years" or "ninety-five years" or "ninety-five-years" or "hundred years" or "hundred-	306575

	years" or "hundred years" or "hundred-years" or "one-hundred years" or "one-hundred-years").ti,ab,kf.	
17	((old* adj3 (subject* or patient*OR participant*)) or (old* adj2 age*) or (old* adj3 adult*)).ti,ab,kf.	213644
18	14 or 15 or 16 or 17	3474815
19	13 and 18	1335
20	exp animals/ not exp humans/	4731219
21	19 not 20	1335
